# Extracts of Salvia-Nelumbinis Naturalis Ameliorate Nonalcoholic Steatohepatitis via Inhibiting Gut-Derived Endotoxin Mediated TLR4/NF-*κ*B Activation

**DOI:** 10.1155/2017/9208314

**Published:** 2017-07-31

**Authors:** Xiangbing Shu, Miao Wang, Hanchen Xu, Yang Liu, Jie Huang, Zemin Yao, Li Zhang

**Affiliations:** ^1^Institute of Digestive Diseases, China-Canada Center of Research for Digestive Diseases (ccCRDD), Longhua Hospital, Shanghai University of Traditional Chinese Medicine, Shanghai 200032, China; ^2^Department of Biochemistry, Microbiology & Immunology, Ottawa Institute of Systems Biology, University of Ottawa, Ottawa, ON, Canada K1H 8M5

## Abstract

Nonalcoholic steatohepatitis (NASH) is featured by the presence of hepatic steatosis combined with inflammation and hepatocellular injury. Gut-derived endotoxin plays a crucial role in the pathogenesis of NASH. Salvia-Nelumbinis naturalis (SNN), a formula of Traditional Chinese Medicine, has been identified to be effective for NASH, but the mechanisms were not thoroughly explored. In the present study, a NASH model was generated using C57BL/6 mice fed a high fat diet (HFD) supplemented periodically with dextran sulfate sodium (DSS) in drinking water for 12 weeks. Mice fed HFD alone (without DSS) or chow diet were used as controls. The NASH mice were given the SNN extracts in the following 4 weeks, while control mice were provided with saline. Mice fed HFD developed steatosis, and DSS supplementation resulted in NASH. The SNN extracts significantly improved metabolic disorders including obesity, dyslipidemia, and liver steatosis and reduced hepatic inflammation, circulating tumor necrosis factor-*α* (TNF-*α*), and lipopolysaccharide (LPS) levels. The beneficial effect of the SNN extracts was associated with restoration of intestinal conditions (microbiota, integrity of intestinal barrier) and inhibition of TLR4/NF-*κ*B activation. These results suggest that the SNN extracts ameliorate NASH progression, possibly through blocking endotoxin related TLR4/NF-*κ*B activation.

## 1. Introduction

Nonalcoholic fatty liver disease (NAFLD) is a main cause of chronic liver diseases, and the global prevalence is approximately 24% [1]. The progressive form of NAFLD has been referred to as nonalcoholic steatohepatitis (NASH). Although NASH represents the minority (10–20%) of patients with NAFLD, it can potentially progress to advanced liver disease leading to cirrhosis, liver-related mortality, and hepatocellular carcinoma (HCC) [2]. Since simple fatty liver is considered to be benign, determination of the risks factors of disease progression is of vital importance. Histological studies identified that the degree of inflammation is the strongest and independent predictor for NAFLD progression [3]. Our current understanding of the pathophysiology of NASH is that excessive fat accumulation coexists with inflammation and cell injury in the liver. Thus, ideal pharmaceutical therapy for NASH should both improve metabolic conditions and target the mechanisms of hepatic cell injury.

NASH is characterized by Kupffer cell activation, and dysbiosis-driven inflammatory plays a vital role [4]. Intestinal microorganisms (endotoxin) produced by opportunistic pathogen in the intestine could enter liver directly through portal vein. These highly conserved molecules known as “pathogen associated molecular patterns” (PAMPs) can be recognized specifically by pattern recognition receptors (PRRs), such as toll-like receptors (TLRs). Studies demonstrated that intraperitoneally injection of LPS, the major component of the outer membrane of Gram-negative bacteria, can exaggerate liver inflammation in high fat diet (HFD) or high calorie diet feeding animals, further indicating the role of gut-derived endotoxin in promoting NASH development [5, 6]. The LPS sensor TLR4 could subsequently trigger a cascade of molecules leading to activation of nuclear factor *κ*B (NF-*κ*B) and production of proinflammatory cytokines and chemokines [7]. In addition, activation of TLRs also attracts other immune cells to the infected sites, thus contributing to the development of NASH.

Prevention and treatment of NASH still confront great obstacles currently. Diet and lifestyle modification are recommended as the first-line therapy. However, these measures cannot be implemented efficiently or maintained in the long run. The need for specific pharmacotherapy is urgent, yet the options available are limited. Recently, natural products have attracted increasing interest in preventing and treating NASH [8]. Herbal medicines derived from Traditional Chinese Medicine (TCM) theories have been applied for treating liver diseases for thousands of years in China. The extracts of Salvia-Nelumbinis naturalis (SNN) formula, initially called Jiangzhi Granula and designed entirely based on TCM theories, have been used to treat NAFLD. We have shown that the SNN extracts can ameliorate NAFLD and related metabolic disorders in patients in a multicenter, randomized, double-blind, placebo-controlled clinical trial [9]. Both in vivo and in vitro experiments in our previous studies confirmed beneficial effects of SNN or its ingredients on insulin resistance and lipid accumulation [10]. Using methionine/choline deficient (MCD) diet-induced NASH model, we have found that the SNN extracts can protect the liver from server damage through improving hepatic antioxidant capability [11]. However, whether SNN extracts can exert a beneficial effect on liver injury related to gut-derived endotoxin has not been determined.

In the present study, we applied NASH mice induced by HFD supplemented with dextran sulfate sodium (DSS) and determined the role of endotoxin in NASH development. With these animals, we assessed the efficacy and potential mechanisms of the SNN extracts in treating of NASH.

## 2. Materials and Methods

### 2.1. Preparation of SNN Extracts

The SNN extracts were prepared as previously described [8]. Briefly, the medicinal materials, 1.5 portion of* Salviae*, 1 portion of* Nelumbinis*, 2.5 portion of* Rhizoma Polygoni Cuspidati,* and 1.5 portion of* Herba Artemisiae Scopariae*, were triturated and blended to powder and then mixed with water/methanol (5 : 95, V/V) for sonication, filtration, and vacuum condensation to obtain the extracts. Methanol was removed from the extracts to gain powder before animal experiments. The main chemical components of the extracts were compared with the previously established standard [12], and 1 g medicinal material can get 200 mg extracts.

### 2.2. Mouse Experiments

Male C57BL/6 mice, 6 weeks of age, were purchased from SLAC Animal Laboratories (Shanghai, China). After one-week acclimatization, the mice were divided into 3 groups: the chow group (*n* = 10) received standard control diet (SLAC Animal Laboratories, Shanghai, China); HFD group (*n* = 6) received HFD (60% of calories derived from fat, Research Diets, NJ, USA); the HF-DSS group (*n* = 26) received HFD supplemented with 1% DSS (MP Biomedicals, Solon, OH, USA) in drinking water. DSS was given in cycles; each cycle consisted of a 7-day DSS administration followed by a 10-day interval with normal drinking water. After 12 weeks of feeding, HFD group mice and 6 mice from the HF-DSS group were sacrificed to evaluate the model. The remaining HF-DSS mice were further divided into 2 groups: HF-DSS group (*n* = 10) remained on HF-DSS diet, while mice in treatment group (*n* = 10) were given the SNN extracts (750 mg/kg) for 4 weeks by gavage.

The body weight and food intake of the mice were recorded at every DSS treatment cycle. The animal protocols were performed in accordance with the guidelines with approval of the Animal Experiment Ethics Committee at Shanghai University of Traditional Chinese Medicine.

### 2.3. Serum Biochemical and Immunological Analysis

After 12 h fasting, mice were anaesthetized with sodium pentobarbital (100 mg/kg) and sacrificed. Blood was collected, and serum triglyceride (TG), total cholesterol (TC), low density lipoprotein cholesterol (LDL-c), high density lipoprotein cholesterol (HDL-c), alanine transaminase (ALT), aspartate transaminase (AST), alkaline phosphatase (ALP), and lactate dehydrogenase (LDH) were analyzed using the Hitachi full-automatic system. Serum LPS and tumor necrosis factor-*α* (TNF-*α*) were detected by ELISA kits (Westang Testmart) according to the manufacturer's instructions.

### 2.4. Hepatic Lipid Content Analysis

Hepatic TG and TC contents were qualified as described previously [8]. Briefly, liver tissue (200 mg) was homogenized in 3 ml of ethanol-acetone (1 : 1) mixture. The homogenate was extracted over night at 4°C and centrifuged for 15 min at 3,000 rpm at 4°C. The organic layer was collected, and TG and TC were qualified using commercial kits (Jiancheng tech, Nanjing, China).

### 2.5. Histology and Immunohistochemistry Analysis

The liver and colon tissues were fixed in 10% formaldehyde, and paraffin-embedded sections (4 *μ*m thickness) were prepared for H&E staining. Frozen liver sections (8 *μ*m thickness) were fixed with 10% paraformaldehyde at room temperature for 30 min and stained with Oil Red O (Sigma, St. Louis, MO) for 60 min. Immunohistochemical staining (IHC) with F4/80 (1 : 200, Abcam, Cambridge, UK) was performed on 4 *μ*m thick paraffin-embedded liver sections following the manufacturer's protocol. Images were captured using Olympus IX71 Inverted microscope (Tokyo, Japan).

### 2.6. Fecal DNA Extraction, Pyrosequencing, and Bioinformatics Statistics

Fresh feces were collected from the ileocecal region of the mice, and genomic DNA was extracted by QIAamp DNA stool mini kit (Qiagen, Germany) as previously described [13]. Purity was determined and concentration was calculated. The extracted DNA was used as the template to amplify the V3 region of 16S rDNA genes. PCR reaction, pyrosequence, and quality control were performed as described previously [13]. The high-quality valid reads were clustered into operational taxonomic units (OTUs) using Mothur (http://www.mothur.org/). Rarefaction curve analysis and Shannon diversity index were analyzed according to the representative sequences of OTUs. A heat map was generated by R software (http://www.R-project.org). Taxonomy-based analysis was performed using the Ribosomal Database Project (RDP) classifier.

### 2.7. Cell Culture and Treatment

Kupffer cells were isolated from pathogen-free male C57/BL6 mice (6–8 weeks, weighing 20 ± 0.5 g) as previously described [14], and their identity was authenticated by the engulfment of immunofluorescence beads. After a 24 h recovery period from isolation, the primary Kupffer cells were cultured in the absence or presence of 50, 100, and 200 ng/ml of LPS (Sigma, St. Louis, MO) for 1, 2, and 4 h.

### 2.8. Quantitative Real-Time PCR

Total RNA was extracted from the liver or LPS treated Kupffer cells using a TRIzol reagent (Invitrogen Corp, Carlsbad, CA, USA) and reversely transcribed into cDNA using reverse transcription kits (Promega, Madison, WI, USA). Sequences of the primers (obtained from Shine Gene, Shanghai, China) used in the experiments were shown in Table 1. Quantitative real-time PCR (qRT-PCR) was performed using the SYBR Green PCR Master Mix kit (TOYOBO, Osaka, Japan) according to the manufacturer's protocol. Quantification of the mRNA concentrations was carried out using the AB StepOnePlus Real-Time PCR System (Applied Biosystems, Carlsbad, CA, USA). The relative mRNA levels were normalized using GAPDH as an internal control and expressed as fold change relative to the control.

### 2.9. Western Blot Analysis

Primary antibodies used for Western blot analysis were anti-zonula occludens 1 (ZO-1), anti-occludin, anti-claudin-1 (Thermo Scientific, Rockford, USA), anti-phosphorylated p65, anti-phosphorylated I*κ*B, anti-p65, anti-I*κ*B (Cell Signaling Technology, Danvers, USA), anti-TIRAP (Toll-interleukin 1 receptor domain containing adaptor protein), anti-IRAK1 (interleukin-1 receptor-associated kinase 1), anti-IRAK4 (Proteintech, Wuhan, China), anti-TRAF6 (TNF receptor-associated factor 6), anti-TLR4 (Abcam, Cambridge, MA, USA), anti-*β*-actin, and anti-Histone H3 (Hua'an Biological Technology, Hangzhou, China). The antibody-antigen complexes were visualized by using the ECL (Electrochemiluminescence) kit (Millipore, Billerica, USA). The intensity of the immunoreactive bands was semiquantified using the GeneTools (SynGene, Frederick, USA).

### 2.10. Statistical Analysis

SPSS 18.0 and GraphPad Prism 5 were used for data analysis. Data were expressed as means ± standard error (SE). One-way analysis of variance (ANOVA) with Tukey's correction was applied for differences between two groups, and *P* < 0.05 was accepted as statistically significant.

## 3. Results

### 3.1. Intestinal Damage Promotes Steatohepatitis

**Figure 1:**
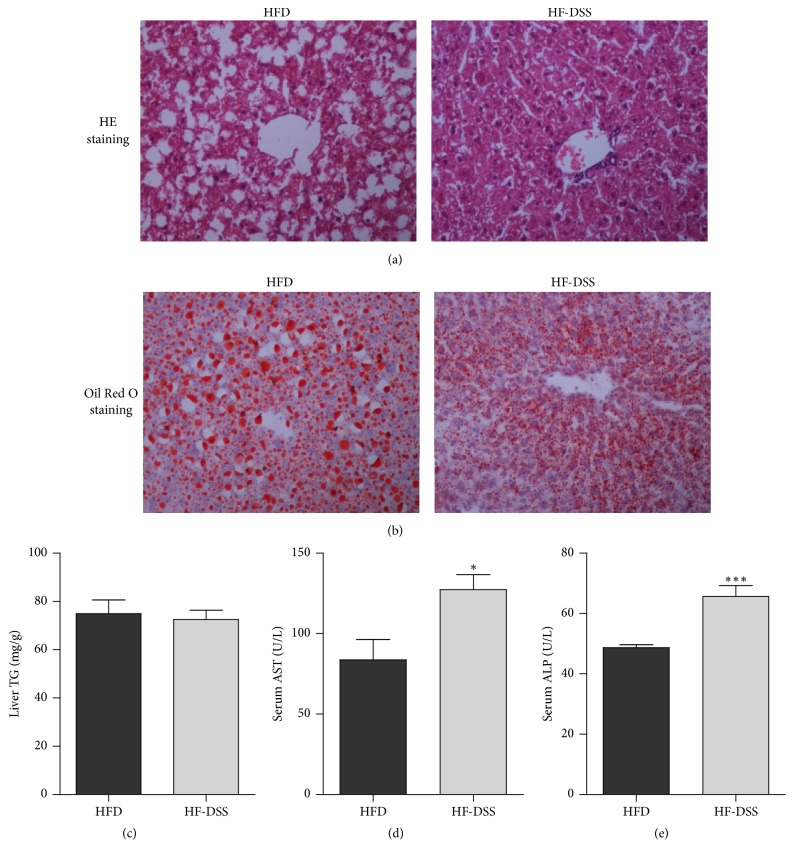
*DSS supplementation promotes steatohepatitis*. Male C57Bl/6 mice (7 weeks of age) were either fed HFD supplemented periodically with DSS in drinking water (*n* = 6) or HFD alone (*n* = 6) for 12 weeks. The mice were sacrificed, and liver tissue and serum were collected. Liver sections were stained with H&E (a) and Oil Red O (b). Image magnification ×200. TG contents in the liver (c), serum AST (d), and ALP (e) were analyzed. Data were present as mean ± SE, ^*∗*^*P* < 0.05, ^*∗∗∗*^*P* < 0.001 versus HFD mice.

Male C57Bl/6 mice fed 12-week HFD exhibited obvious hepatic steatosis but no sign of inflammation in liver sections (Figure 1(a)). Supplementation of HFD with DSS, however, resulted in damage in the intestinal barriers, which was associated with significant hepatic steatosis combined with inflammation foci (Figures 1(a) and 1(b)). While the liver TG contents were comparable between the two groups of mice (Figure 1(c)), the HF-DSS mice presented increased level of serum AST (Figure 1(d)) and ALP (Figure 1(e)), suggesting that intestinal damage might have contributed to the development of NASH.

### 3.2. The SNN Extracts Treatment Alleviated Hepatomegaly in NASH Mice

NASH mice developed hepatomegaly and exhibit increased body weight (Table 2); the ratio of liver/body weight of NASH mice was not different from that in control mice fed a normal chow diet (Table 2). After 4-week SNN treatment, body weight, liver weight, and liver/body weight ratio were all significantly decreased compared with those of untreated NASH mice (Table 2). Because food intake between untreated and SNN-treated groups was identical (Table 2), the beneficial effect of the SNN extracts on body weight and liver weight was unlikely related to changes in satiety.

### 3.3. The SNN Extracts Improved Serum Lipid Profiles and Enzymes in NASH Mice

**Figure 2:**
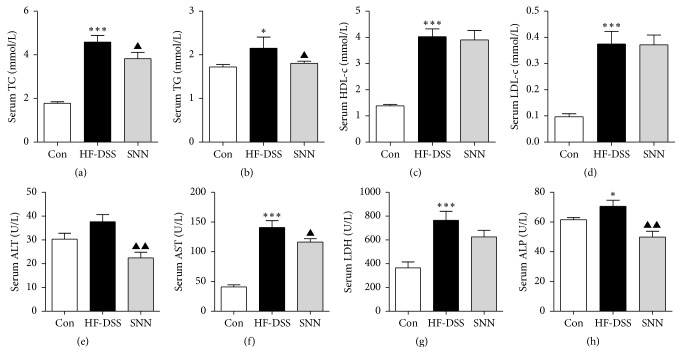
*The effect of SNN extracts on serum lipid profiles and enzymes*. Male C57Bl/6 mice (7 weeks of age) were fed HF-DSS diet for 12 weeks followed by either 4-week SNN (*n* = 10) or normal saline supplementation via gavage (*n* = 10), while chow diet mice were set as controls (Con, *n* = 8). The mice were sacrificed, blood was collected, and serum was separated. Serum TC (a), TG (b), HDL-c (c), and LDL-c (d) were analyzed; enzymes ALT (e), AST (f), LDH (g), and ALP (h) in serum were detected. Data were present as mean ± SE, ^*∗*^*P* < 0.05, ^*∗∗∗*^*P* < 0.001 versus Con mice; ^▲^*P* < 0.05, ^▲▲^*P* < 0.01 versus HF-DSS (NASH) mice.

NASH mice developed hyperlipidemia after 16 weeks of HF-DSS feeding. Thus, the serum TC, TG, and LDL-c concentrations were increased as compared to that in chow diet fed controls (Figure 2). Treatment of mice with the SNN extracts for 4 weeks significantly reduced the serum TG concentration, whereas the serum TC concentration was restored to normal as compared to that in untreated NASH mice (Figures 2(a) and 2(b)). Unexpectedly, the level of HDL-c was significantly increased in NASH mice, while there were no differences in HDL-c between the SNN-treated and untreated groups (Figure 2(c)).

Liver enzymes (AST, ALP, and LDH) were significantly increased in mice fed HF-DSS diet, and the SNN extract treatment resulted in markedly decreased serum AST and ALP in these mice (Figures 2(f)–2(h)). There was no difference in serum ALT between NASH mice and chow diet fed mice. However, the SNN extract treated mice had lowered serum ALT compared with that in untreated NASH mice (Figure 2(e)).

### 3.4. The SNN Extracts Attenuated Hepatic Steatosis in NASH Mice

**Figure 3:**
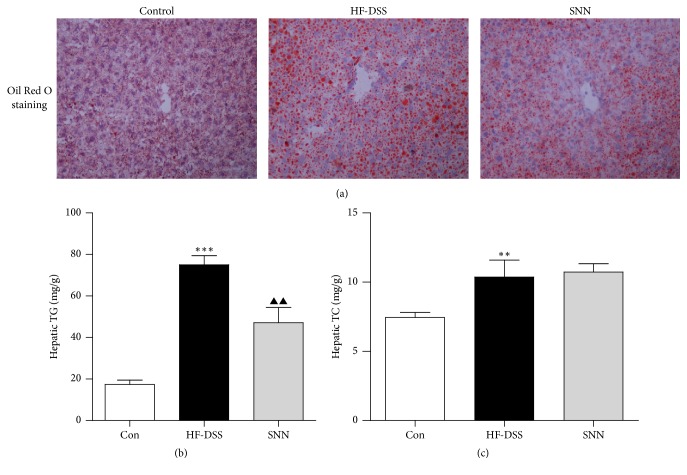
*The effect of SNN extracts on hepatic steatosis*. Male C57Bl/6 mice (7 weeks of age) were fed HF-DSS diet for 12 weeks followed by either 4-week SNN (*n* = 10) or normal saline supplementation via gavage (*n* = 10), while chow diet mice were set as controls (Con, *n* = 8). The mice were sacrificed, and liver tissues were collected and stained with Oil Red O (a). Image magnification ×200. Hepatic TG (b) and TC (c) were analyzed. Data were present as mean ± SE, ^*∗∗*^*P* < 0.01, ^*∗∗∗*^*P* < 0.001 versus Con mice; ^▲▲^*P* < 0.01 versus HF-DSS (NASH) mice.

Upon HF-DSS dieting, mice developed obvious hepatic steatosis as demonstrated by histologic analysis using Oil Red O staining of the liver sections (Figure 3(a)). Increased hepatic TG and TC contents were in accordance with the observed histologic changes (Figures 3(b) and 3(c)). The hepatic steatosis was attenuated upon the SNN extracts treatment. Likewise, hepatic TG content was also markedly reduced in mice treated with the SNN extracts (Figure 3(b)). However, the SNN extracts treatment had no effect on hepatic TC concentration in NASH mice (Figure 3(c)).

### 3.5. SNN Treatment Ameliorated Liver Inflammation in NASH Mice

**Figure 4:**
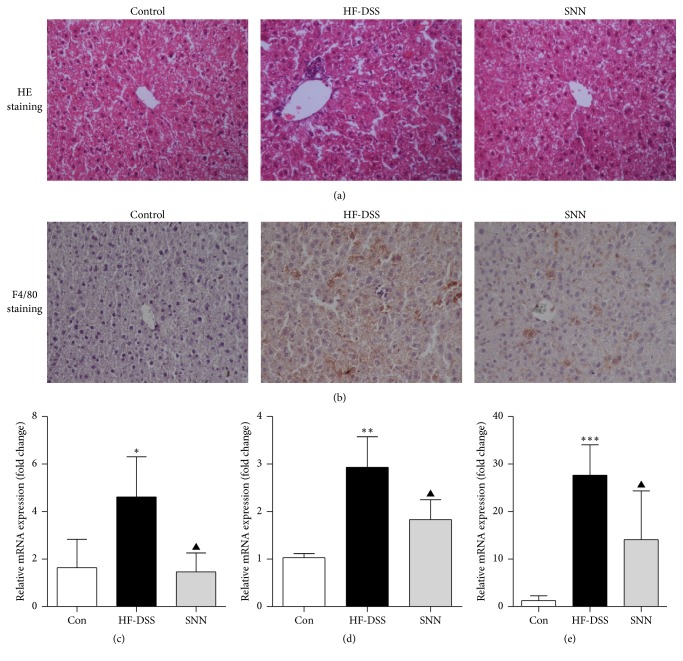
*The effect of SNN extracts on liver inflammation*. Male C57Bl/6 mice (7 weeks of age) were fed HF-DSS diet for 12 weeks followed by either 4-week SNN (*n* = 10) or normal saline supplementation via gavage (*n* = 10), while chow diet mice were set as controls (Con, *n* = 8). The mice were sacrificed, liver tissues were collected and stained with HE (a), and Kupffer cell activation was indicated by the F4/80 IHC (b). Image magnification ×200. Hepatic IL-6 (c), MCP-1 (d), and TNF*α* (e) mRNA was qualified by qRT-PCR. Data were present as mean ± SE, ^*∗*^*P* < 0.05, ^*∗∗*^*P* < 0.01, and ^*∗∗∗*^*P* < 0.001 versus Con mice; ^▲^*P* < 0.05 versus HF-DSS (NASH) mice.

NASH mice developed hepatic steatosis and inflammation, and both of which were alleviated by the 4-week SNN extracts gavage (Figure 4(a)). Immunohistochemical (IHC) staining of liver sections of NASH mice showed that expression of F4/80, the membrane protein and an indicator of activated Kupffer cells, was decreased after the SNN extract treatment (Figure 4(b)). The mRNA concentrations of monocyte chemotactic protein 1 (MCP-1), IL-6, and TNF-*α* in the liver were significantly increased in NASH mice, and the increase was largely blocked by SNN extracts treatment (Figures 4(c)–4(e)).

### 3.6. Effect of the SNN Extracts Treatment on Gut Microbiota in NASH Mice

**Figure 5:**
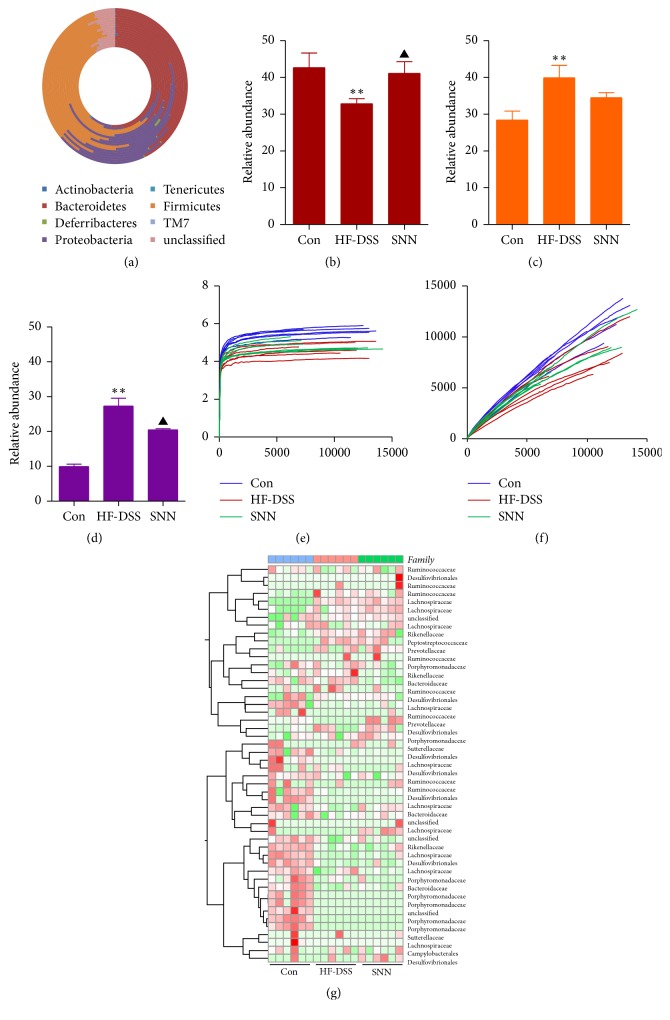
*The effect of SNN on the composition of gut microbiota*. Fresh feces were collected from the ileocecal region of the mice, and taxonomic structure of 16s rRNA gene was assessed using the Illumina MiSeq platform (*n* = 6 per group) and the species at phylum level were demonstrated (a); the alterations of Firmicutes (b), Bacteroidetes (c), and Proteobacteria (d) were indicated. Bacterial diversity was shown by Shannon curves (e) and Refraction curves (f). Key OTUs indicating genus-level changes based on the genus composition and abundance were generated (g). The relative abundance of each genus was indicated by a gradient of color from green (low abundance) to red (high abundance). Data were present as mean ± SE, ^*∗∗*^*P* < 0.01 versus Con mice; ^▲^*P* < 0.05 versus HF-DSS (NASH) mice.

We performed 16s rDNA analysis using Illumine MiSeq and detected seven dominant phyla in the mouse feces, namely, Bacteroidetes, Firmicutes, Proteobacteria, Tenericutes, Deferribacteres,* TM7,* and Actinobacteria (Figure 5(a)). In NASH mice, the relative abundance of Firmicutes was significantly decreased and that of Bacteroidetes and Proteobacteria was significantly increased as compared to that in the control mice (Figures 5(b)–5(d)). Treatment with the SNN extracts for 4 weeks resulted in partial normalization of the decreased abundance of Firmicutes and increased abundance of Proteobacteria (Figures 5(b) and 5(d)). However, the bacterial diversity of the gut microbiota was not affected by the SNN extract treatment as suggested by the Shannon-Wiener curves (Figure 5(e)) and rarefaction curves (Figure 5(f)).

The major bacterial families identified in our analysis are shown in the hierarchical clustering heat map (Figure 5(g)). Altogether, 12 altered bacterial families were detected in NASH mice. With the SNN extract treatment, the relative abundance of Ruminococcaceae and Lachnospiraceae was increased, whereas the relative abundance of Desulfovibrionales and Campylobacterales was reduced in comparison to that in untreated NASH mice (Figure 5(g)).

### 3.7. The SNN Extracts Reduced Intestinal Injury and Blocked LPS Release

**Figure 6:**
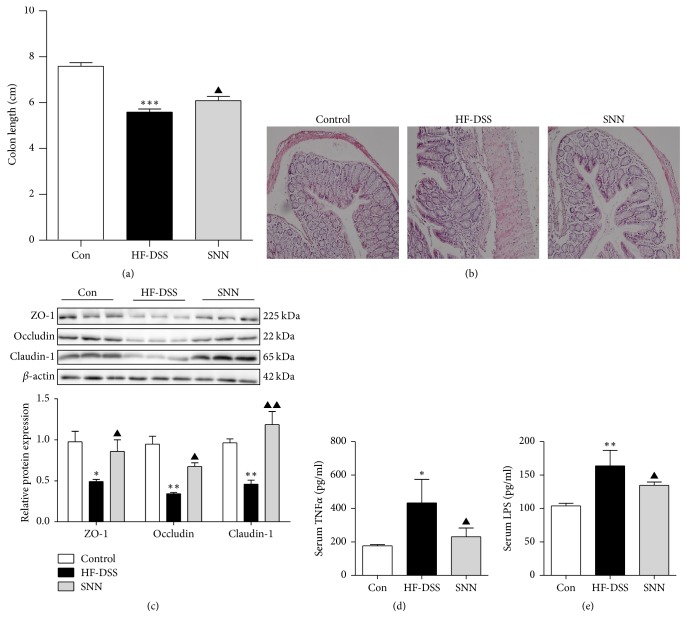
*The effect of SNN on intestinal injury and LPS release*. The colons of the mice were collected, the length was recorded (a), and the colon sections were further stained with HE (b). TJ proteins including ZO1, Occludin, and Claudin 1 were analyzed by Western blot (c). Circulating TNF*α* (d) and LPS (e) levels were detected by ELISA kits. Original magnification of representative images, ×200; data were present as mean ± SE, ^*∗*^*P* < 0.05, ^*∗∗*^*P* < 0.01, and ^*∗∗∗*^*P* < 0.001 versus Con mice; ^▲^*P* < 0.05, ^▲▲^*P* < 0.01 versus HF-DSS (NASH) mice.

Gut-derived endotoxin may enter the circulation through damaged intestinal barrier. We next examined the integrity of the colon in NASH mice. Significant colon shortening was observed in these mice (Figure 6(a)), and architectural disruption of the crypts, increased severity of epithelial damage, and increased inflammation were detected in the colon sections (Figure 6(b)). Remarkably, the SNN extracts treatment was shown to protect the colons from this damage (Figures 6(a) and 6(b)). Further determination of proteins responsible for the integrity of intestinal barrier showed that the SNN extract treatment significantly increased the levels of ZO1, occludin, and claudin-1 (Figure 6(c)). Moreover, the elevated LPS and TNF-*α* levels observed in the NASH mice could be significantly attenuated by the SNN extracts treatment (Figures 6(d) and 6(e)).

### 3.8. LPS Aroused Inflammation in Kupffer Cells

**Figure 7:**
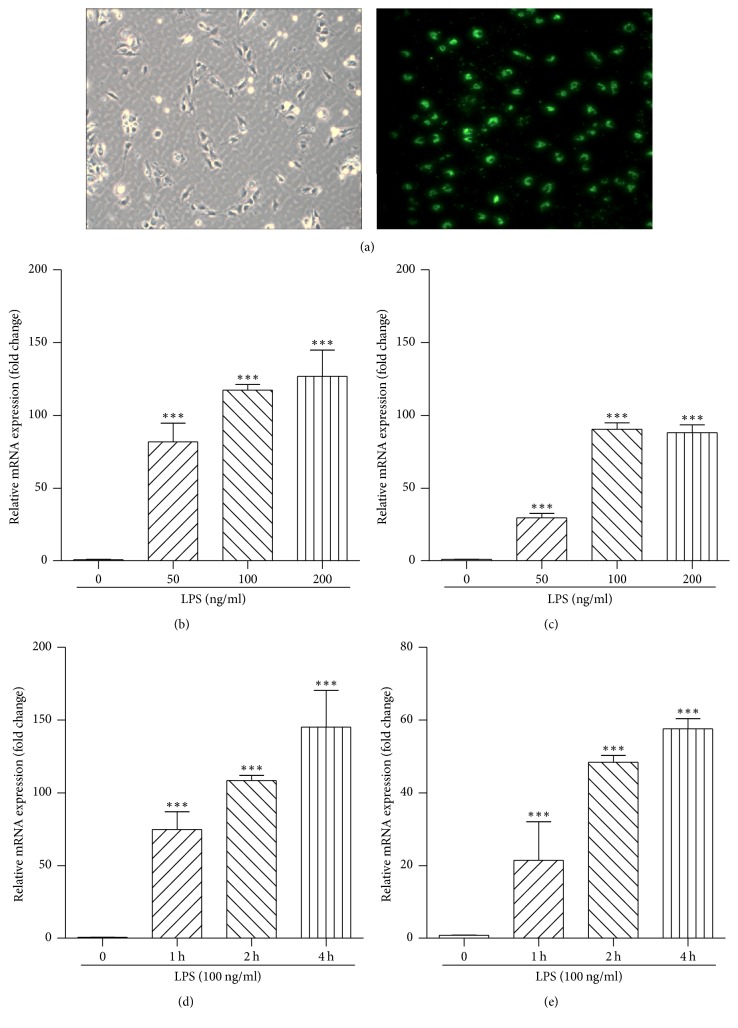
*LPS induced inflammation in Kupffer cells*. Kupffer cells were isolated from C57BL/6 mice and identified by fluorescently labeled beads (a). Kupffer cells were treated with LPS (50, 100, and 200 ng/ml) for 2 h, and mRNA expression of TNF*α* (b) and IL-*β* (c) in the cells was qualified by qRT-PCR. Kupffer cells were treated with LPS (100 ng/ml), and the cells were collected at different time points (1 h, 2 h, and 4 h). Cellular TNF*α* (d) and IL-*β* (e) mRNA expression was analyzed by qRT-PCR. Original magnification of representative images, ×200; data were present as mean ± SE, ^*∗∗∗*^*P* < 0.001 versus cells cultured with routine medium.

We performed additional in vitro experiments to ascertain that LPS is responsible for the inflammatory response of hepatic macrophages. To this end, Kupffer cells were isolated from the mouse liver, and their phagocytic activity was validated by engulfment of fluorescently labeled beads (Figure 7(a)). Incubation Kupffer cells with LPS resulted in marked increase in TNF-*α* and IL-*β* mRNA, in a dose- (Figures 7(b) and 7(c)) and time-dependent (Figures 7(d) and 7(e)) manner. These in vitro data provide indirect support to the above in vivo observation (Figure 4) and suggest that gut-derived endotoxin may elicit proinflammatory response in the liver (through Kupffer cells).

### 3.9. SNN Treatment Regulated the TLR Signaling Pathway

**Figure 8:**
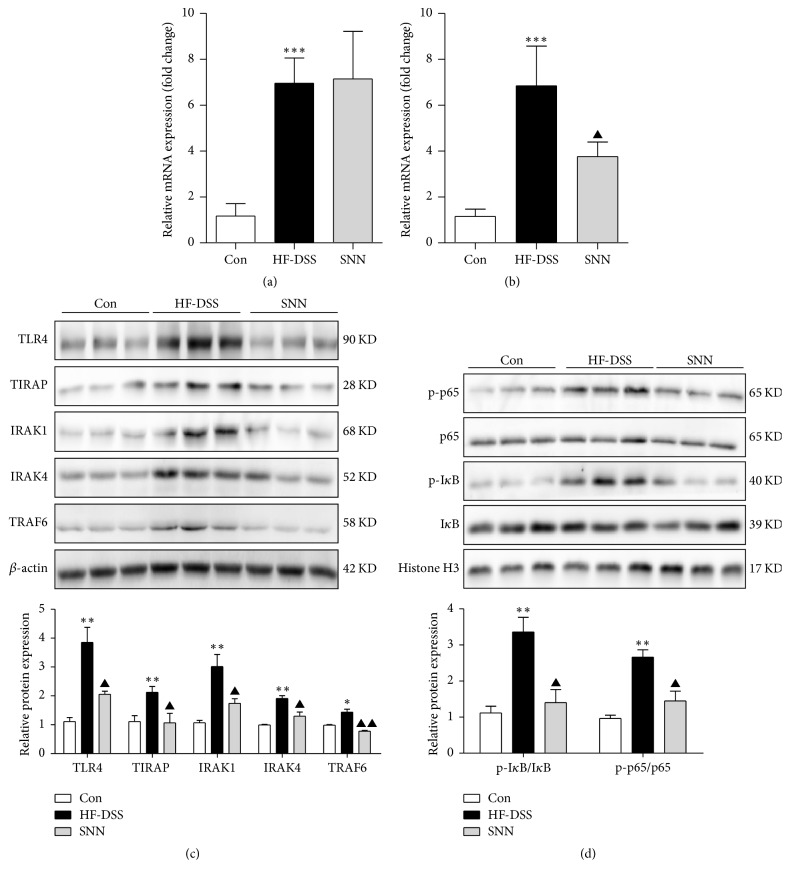
*The effect of SNN on TLR4 mediated NF-κB activation*. The livers of the mice were collected, and the mRNA expressions of hepatic TLR2 (a) and TLR4 (b) were analyzed by qRT-PCR, and the key molecules in TLR4/NF-*κ*B pathway were detected by Western blot (c and d). Data were presented as mean ± SE,* n* = 3–6, ^*∗*^*P* < 0.05, ^*∗∗*^*P* < 0.01, and ^*∗∗∗*^*P* < 0.001 versus Con mice; ^▲^*P* < 0.05, ^▲▲^*P* < 0.01 versus HF-DSS (NASH) mice.

Among the twelve functional TLRs present in mice, TLR2 and TLR4 are abundantly expressed in the liver. The mRNA of TLR2 and TLR4 was significantly increased in NASH mice. The TLR4 mRNA (Figure 8(a)) was decreased in the SNN extract treated mice, whereas the TLR2 mRNA was unchanged (Figure 8(b)). These results suggested that the SNN extracts might diminish the endotoxin effect.

Finally, we determined the effect of SNN extracts on the levels of key molecules involved in the TLR4/NF-*κ*B pathway, namely, TLR4, TIRAP, IRAK1/4, and TRAF6. We found that the SNN extracts almost completely (e.g., TIRAP, TRAF6) or partially (e.g., TLR4, IRAK1, and IRAK4) restored the altered protein concentrations that occurred in the NASH mice (Figure 8(c)). Further analysis of members of the NF-*κ*B transcription factor family (such as p65) and their phosphorylate status showed increased phosphorylation of I*κ*B and p65 in the livers of NASH mice, and the SNN extracts blunted I*κ*B and p65 phosphorylation (Figure 8(d)). These results together suggest that the SNN extracts can inhibit TLR4/NF-*κ*B activation.

## 4. Discussion

NASH is becoming the leading cause of chronic liver diseases and could result in an increase in the overall and liver-related mortality. In the present study, we demonstrated that the herbal medicine formula SNN inhibited the release of gut-derived endotoxin and blocked TLR4 mediated NF-*κ*B activation in a mouse model of NASH, thus demonstrating the protective effect of SNN on NAFLD progression.

Accumulating evidence indicates that gut microbiota is associated with the development of NASH [15, 16]. It is reported that NAFLD patients, and in particular those with NASH, are more likely to have increased intestinal permeability compared with healthy controls [17]. Intervention of intestinal microbiota with antibiotic or prebiotics has been proved to be beneficial for NAFLD/NASH patients, indicating that the change of intestinal environment could affect NAFLD development and progression [18, 19]. Imbalances in the structure of the gut microbiota induced by HFD consumption may impair the integrity of gut barrier and increase the levels of endotoxin in liver through the portal vein [20]. Therefore, the gut microbiota represents a potential target of therapeutic drugs or nutritional interventions. We have identified SNN which acted on restoring the increase of opportunistic pathogens. Prebiotics or probiotics are reported to selectively modulate of the structure of gut microbiota, thus contributing to the improvement of intestinal function. The natural product berberine has been proved to prevent metabolic conditions (i.e., obesity, insulin resistance, and type 2 diabetes) in animals and patients, and gut microbiota modulation is thought to be the key mechanism.

Intestinal permeability is regulated by tight junctions (TJ); among the identified TJ, the transmembrane proteins occludin, claudins, and cytoplasmic proteins zonula occludens 1 (ZO-1) are considered crucial in regulating intestinal permeability [21]. Dysfunction of intestinal barrier can facilitate the hepatic entrance of intestinal microorganisms including LPS [6]. LPS is a component of the outer wall of Gram-negative bacteria, and itself can induce the intestinal barrier dysfunction and epithelial cells injury. We have compared 12-week HF-DSS diet with HFD feeding to the mice and confirmed that increased intestinal permeability accelerates the development of NASH, indicating that endotoxin release to liver was a potential risk factor for liver inflammation.

Circulating levels of gut-derived LPS are increased in NAFLD patients [22]. We have isolated Kupffer cells from the mice, incubated the cells along with LPS, and detected increased TNF-*α* and IL-1*β* expression in time and dose dependent manner. Our results were consistent with reports which showed that injection of LPS to mice can induce inflammatory response in the liver [23, 24].

The innate immune response provides the first line of host defense against invading pathogens. This response is triggered by the activation of PRRs. Among the growing family of PRRs, TLRs play a fundamental role in the primary response against invaders [7]. The specific detection of PAMPs and DAMPs by host receptors drives a cascade of signaling that converges at NF-*κ*B and induces the secretion of proinflammatory cytokines. The activation of NF-*κ*B typically involves phosphorylation of I*κ*B by the inhibitor of nuclear factor-*κ*B kinase (IKK) complex. The phosphorylation of I*κ*B leads to its ubiquitylation and subsequent degradation, which allows the release of NF-*κ*B and its translocation to the nucleus. The most common heterodimer of NF-*κ*B is P65/P50 complex; subsequent to its translocation, P65 undergoes site-specific posttranslational modifications to further enhance the function [25]. We have analyzed the key molecules in the cascade pathway and found that SNN could inhibit the protein expression of TLR4, TIRAP, IRAK1/4, and TRAF6, which facilitate the phosphorylation of I*κ*B and activation of NF-*κ*B. These changes were consistent with the alteration of cytokines in both serum and liver tissues, further indicating that SNN specifically blocks LPS related hepatic inflammation. Suppression of LPS/TLR4/NF-*κ*B pathway has been reported to be beneficial for inflammatory diseases, and many natural products, such as berberine, curcumin, and resveratrol, have been identified to be potential inhibitors of such cascade [26–28].

TLRs act as a double-edged sword: deficient TLR signaling might render the organism vulnerable to exposure to pathogenic attack, while an excessive TLR response, such as activation of TLR4 on the Kupffer cells, results in uncontrolled release of a range of proinflammatory cytokines and chemokines [29]. SNN is a safe therapeutic agent because it has been used for decades in China. Our previous basic research and clinical trial also showed that SNN is safe and effective in treating rodents and patients with NAFLD [10, 11]. However, since gut microbiota alteration could induce LPS, while chemically intestinal damage accelerates LPS release to the liver, the restored TLR4/NF-*κ*B pathway on the SNN extracts treatment might be secondary to the blockage of LPS release from the intestine.

## 5. Conclusions

In summary, we identified that intestinal damage could accelerate the development of NASH, and the herbal medicine formula, SNN, significantly attenuated liver steatosis and inflammation in experimental mice. By improving intestinal environment and hepatic endotoxin entrance, SNN acts on TLR4/NF-*κ*B pathway associated with NASH pathological progression. Our findings supported a beneficial role of SNN and indicated that SNN might be an effective therapeutic strategy against NAFLD progression.

## Figures and Tables

**Table 1:** Sequences of the primers used for PCR.

Genes	Forward primer	Reverse primer
TNF*α*	CCCTCCAGAAAAGACACCATG	CACCCCGAAGTTCAGTAGACAG
IL1*β*	GCTTCAGGCAGGCAGTATCA	TGCAGTTGTCTAATGGGAACG
IL-6	GGGACTGATGCTGGTGACAAC	CAACTCTTTTCTCATTTCCACGA
MCP-1	GCTGACCCCAAGAAGGAATG	TTGAGGTGGTTGTGGAAAAGG
TLR2	TTCACCACTGCCCGTAGATG	GGTACAGTCGTCGAACTCTACCTC
TLR4	TTACACGTCCATCGGTTGATC	TACACCTGCCAGAGACATTGC
GAPDH	GTGCCGCCTGGAGAAACC	GGTGGAAGAGTGGGAGTTGC

**Table 2:** The change of body weight, liver weight, and food intake of the mice.

Parameters	Control	HF-DSS	SNN
Body weight (g)	28.5 ± 0.64	32.5 ± 1.51^*∗∗*^	27.9 ± 1.56^#^
Liver weight (g)	1.3 ± 0.03	1.5 ± 0.04^*∗∗*^	1.2 ± 0.07^##^
Liver/body weight (%)	4.6 ± 0.06	4.5 ± 0.15	4.0 ± 0.10^#^
Food intake (g/d)	11.9 ± 0.23	7.9 ± 0.38^*∗∗∗*^	8.0 ± 1.07

*n* = 10 per group, ^*∗∗*^*P* < 0.01, ^*∗∗∗*^*P* < 0.001 versus control group; ^#^*P* < 0.05, ^##^*P* < 0.01 versus HF-DSS group.
